# The association between toe grip strength and osteoarthritis of the knee in Japanese women: A multicenter cross-sectional study

**DOI:** 10.1371/journal.pone.0186454

**Published:** 2017-10-17

**Authors:** Daisuke Uritani, Takahiko Fukumoto, Tomoki Myodo, Kazuhito Fujikawa, Miyuki Usui, Daisuke Tatara

**Affiliations:** 1 Department of Physical Therapy, Faculty of Health Science, Kio University, Nara, Japan; 2 Department of Rehabilitation, Nishinokyo Hospital, Nara, Japan; 3 Department of Rehabilitation, Yamato Kashihara Hospital, Nara, Japan; 4 Department of Rehabilitation, Oogaki Chuo Hospital, Gifu, Japan; 5 Department of Rehabilitation, Fukuoka Shion Hospital, Fukuoka, Japan; University of Umeå, SWEDEN

## Abstract

We evaluated the relationship between altered foot function and knee osteoarthritis through a specific comparison of toe grip strength in older women with and without knee osteoarthritis. Participants were 120 women with knee osteoarthritis (OA group) and 108 healthy community-dwelling women with no history of knee pain and lower limb surgery (control group). The following factors and outcome variables were included in our analysis: measured toe grip strength, isometric knee extension strength, the timed Up-and-Go, and the WOMAC scale. Between-group differences were compared using unpaired *t*-tests for descriptive characteristics and outcome measures. Pearson’s correlation coefficients between toe grip strength and age, body mass index, and other outcome measures were calculated. Multiple logistic regression analysis was used to evaluate the independence of toe grip strength and knee osteoarthritis. Compared to the control group, participants in the OA group were older, heavier, and had a higher body mass index. Toe grip strength and isometric knee extension strength were lower and the timed Up-and-Go longer for the OA group than the control group. Toe grip strength was correlated with age negatively and isometric knee extension strength positively in the OA group and to age and the timed Up-and-Go negatively, and isometric knee extension strength positively in the control group. Multiple logistic regression analysis revealed that body mass index, isometric knee extensor strength, and toe grip strength were independently associated with knee osteoarthritis. Our findings indicate a probable association between altered forefoot function and the incidence or progression of knee osteoarthritis. Increasing toe grip strength might provide a practical intervention for patients with knee osteoarthritis.

## Introduction

Knee osteoarthritis (OA) is one of the most common conditions leading to decreased physical activity and increased disability in older adults globally [1, 2]. High mechanical stress exerted on the joint surfaces is the one of the important risk factors for the development and progression of knee OA [3–6]. Abnormal loading of the knee joint can also result from an altered relationship between the kinematics of the foot and knee [7, 8].

Gross et al. [9] reported that the association between a pes planus and cartilage damage of the medial tibiofemoral joint compartment is not dependent on the presence of a varus knee malalignment. Moreover, they stated that the association between pes planus and frequent knee pain might be similarly independent of the presence of either varus or valgus knee alignment [9]. Overall, Gross et al. argued that the result in their study indicated that changes of foot structures’ characteristics led to knee pathology, while they did not support the reverse causation that knee OA leads to knee joint malalignment and a subsequent pes planus morphology as a compensatory posture [9]. Therefore, abnormal foot posture or altered foot kinematics may be one of the reasons for degenerative change of the knee joint cartilage due to abnormal loading of the knee.

Although the association between foot posture and altered mechanics of the knee joint in OA has been investigated by several authors [7, 8, 10–14], the actual toe kinematics and function in individuals with knee OA has received little attention. Of specific interest is the effect of toe flexor function, which is important for walking [15, 16], on knee OA. Toe flexion during terminal stance is a key component of the windlass mechanism, which supports the rigid supination of the foot for push-off that is required for smooth progression of the body during walking [17]. Yet, the inability of patients with knee OA to maintain contact of the toes with the ground over the stance phase of gait until push-off is a frequent observation in clinical practice, indicative of a possible association between knee OA and an impaired function of toe flexors during walking.

As the mechanics of the forefoot and the hallux are functionally linked to the mechanics of the rearfoot via the midfoot [18], dysfunction of the hallux may indirectly influence the altered kinematics of the knee joint in individuals with knee OA through its effects on the kinematics of the rearfoot during walking [8–14]. Dananberg also described the association between impairment in the range of motion of the hallux and alterations in the mechanics of the foot and motion of the lower leg during walking [19], which may lead to compensatory malalignment of the knee [20, 21].

Based on this evidence, we inferred that decreasing toe flexor function may be associated with knee joint pathology. Therefore, the aim of our study was to evaluate the association between TGS and knee OA through a comparison of TGS in Japanese women with and without knee OA. We hypothesized that a decrease in toe grip strength (TGS), which is one of the toe flexor functions, would be associated with the incidence of knee OA.

## Methods

### Participants

Participants were 120 women (69.4±3.6 years old) with knee OA (OA group) and 108 healthy community-dwelling women (67.3±3.6 years old) without complaints of knee pain (control group). Participants in the OA group were recruited from 4 hospitals located in different regions of Japan. Data were collected between April 2013 and January 2014. The OA group consisted of both outpatients receiving conservative treatment for their knee OA and inpatients awaiting total knee arthroplasty (TKA). Regarding inpatients, we measured their data within a few days from hospital admission. Participants in the OA group were further screened to exclude those with known neuromuscular or musculoskeletal pathologies; who had undergone a TKA or any other surgical treatment of the lower limbs or trunk; and those with health conditions, which could influence the function of the foot, including neurological or orthopedic impairments and diabetes mellitus. The control group consisted of volunteers who participated in municipal surveys on physical fitness, being conducted and managed by two different municipalities in Japan. Individuals in the control group were screened by a physical therapist to ensure absence of any knee pain and limited knee range of motion, or malalignment for both sides. Participants in the control group were also further screened to exclude those with known neuromuscular or musculoskeletal pathologies; who had undergone any surgical treatment of the lower limbs or trunk; and those with health conditions, which could influence the function of the foot, including neurological or orthopedic impairments and diabetes mellitus through interview by physical therapists. All participants in the control group could walk by themselves without a walking aid, such as cane.

Our study was approved by our institutional Research Ethics Committee (H23-35). Details of the testing procedures and the aim of the study were explained to all participants and written informed consent was also obtained from all participants before study inclusion.

### Experimental procedure

Relevant descriptive characteristics (age, height, weight, and body mass index (BMI)) were extracted from the medical record of patients forming the OA group, and measured for participants in the control group. The severity of knee OA for patients in the OA group was determined based on the Kellgren-Lawrence (KL) grading system [22], with the grade obtained from the medical record. In the OA group only, the Western Ontario and McMaster Universities Osteoarthritis Index (WOMAC; Japanese version LK3.1) was used to evaluate self-reported knee joint pain, stiffness, and limitations in physical function [23]. TGS and isometric knee extension strength (IKES) were measured on the affected side for patients in the OA group, and for the non-dominant side for participants in the control group, where the dominant leg was identified as the one preferred for kicking a ball. In general, it is speculated that non-dominant leg is preferred to sustain weight during one-leg standing, kicking a ball, and stepping up and down,; and is predisposed to exposure to mechanical stress more so than the dominant leg. Hence, we measured lower extremity strength in the non-dominant side.

TGS was measured using a toe-grip dynamometer (T.K.K. 3362, Takei Scientific Instruments, Niigata, Japan), using previously described methods [24, 25]. A previous study reported substantial to almost perfect inter- and intra-rater reliability for this device when used among people 60 to 79 years old [24]. For measurement, participants sat upright on a chair, without leaning on the backrest, with hips and knees flexed at approximately 90°; the ankles were placed in the neutral position and fixed with a strap. The first proximal phalanx was positioned on the grip bar and the heel stopper was individually adjusted to fit the heel for each participant. The first toe was used as the benchmark for establishing the testing position, because the flexor strength of the hallux has been reported to be most strongly associated with total TGS [26]. Before the actual measurements, participants practiced the test at a submaximal effort. For the actual measurements, participants were instructed to grip the bar with their toes exerting the greatest possible force for approximately 3 s. Two TGS measurements were recorded and the mean value was used for analysis.

Isometric knee extension strength (IKES) was measured using a hand-held dynamometer (μ-tas F1, ANIMA, Tokyo, Japan) with participants in a seated position with the knee in 90° of flexion and using previously described methods [27]. Participants were instructed to gradually increase the intensity of knee extension against the dynamometer for approximately 2 s, avoiding an explosive contraction, and to maintain their maximal force output for approximately 3 s. Again, two measurements were obtained, with the mean used for analysis.

The timed Up-and-Go (TUG) was used as a behavioral measure of knee function, using standard test methods[28]. Briefly, participants were instructed to stand up from a seated position in a chair, walk as quickly and safely as possible (without running) towards a pole, turn around the pole, and then walk back to the chair and sit down. The time needed to complete the TUG was recorded using the TUG meter (T.K.K. 5804, Takei Scientific Instruments, Niigata, Japan). Each participant completed the TUG twice, with the mean time used for analysis.

### Data analysis

Between-group differences in descriptive characteristics and outcome measures were evaluated using an unpaired *t*-test. TGS was also compared among each KL grade in the OA group and the control group using one-way analysis of variance (ANOVA) and post-hoc tests. The within-group correlation between TGS and age, BMI, IKES, and the TUG was evaluated using Pearson’s correlation coefficients. The association between TGS and knee OA was evaluated using multiple logistic regression analysis with group assignment as the dependent variable (OA = 1, control = 0) and age, BMI, TGS, and IKES as the independent variables. For all analyses, the significance level was set at 5%. All statistical analyses were performed using SPSS software (version 22.0, SPSS Inc., Chicago, IL).

## Results

The distribution of KL grades among patients in the OA group was as follows: KL grades 2, 3, and 4 in 12, 41, and 64 patients, respectively, with the KL undefined for 3 patients. Descriptive characteristics and outcome measures for the OA and control groups, as well as the ROM and WOMAC subscale scores for the OA group, are summarized in Table 1. The following between-group differences were identified. Compared to the control group, patients in the OA group were older (p<0.05), and had a greater body weight and higher BMI (p<0.01). With regards to measured outcomes, TGS and IKES values were lower for the OA group than the control group (p<0.01), with a slower TUG in the OA group compared to the control group (p<0.01). In addition, mean TGS in all subgroups were also significantly lower than that in the control group (p<0.01).

**Table 1:** Participants’ descriptive characteristics and outcome measures.

	OA group	Control group
**Age, years**	73.3 (7.4)*	71.0 (6.7)
**Height, cm**	150.7 (5.5)	151.7 (5.2)
**Weight, kg**	57.7 (9.9)**	50.8 (7.3)
**BMI, kg/m**^**2**^	25.3 (3.8)**	22.1 (2.8)
**TGS, kg**	6.7 (3.3)**	11.1 (4.8)
**KL2**	7.4 (3.9)^##^	
**KL3**	6.5 (3.1)^##^	
**KL4**	6.6 (3.3)^##^	
**IKES, kg**	13.4 (5.1)**	23.2 (5.9)
**TUG, seconds**	12.4 (4.7)**	5.9 (1.0)
**WOMAC**^**$**^**, points**		
**Pain**	7.8 (3.9)	
**Stiffness**	3.0 (2.1)	
**Physical function**	25.9 (13.6)	

OA; osteoarthritis, BMI; Body mass index, TGS; Toe grip strength, IKES; Isometric knee extension strength, TUG; Timed Up and Go, KL; Kellgren-Lawrence grade, WOMAC; The Western Ontario and McMaster Universities Osteoarthritis Index.

^$^Scores of WOMAC subscale range from 0 to 20 in pain, from 0 to 8 in stiffness, and 0 to 68 in physical function, with a higher score indicating more severe disease.

Data were expressed as mean (standard deviation).

*p<0.05

**p<0.01

^##^p<0.01 vs mean value of TGS of the control group based on one-way analysis of variance.

TGS was significantly correlated to age (r = -0.31) and IKES (r = 0.40) in the OA group and with age (r = -0.65), IKES (r = 0.52), and the TUG (r = -0.48) in the control group (Table 2).

**Table 2:** Correlation coefficient between TGS and participants’ characteristics.

	Age	BMI	IKES	TUG
**OA group**	-0.31**	0.07	0.40**	-0.23
**Control group**	-0.65**	0.05	0.52**	-0.48**

TGS; Toe grip strength, BMI; Body mass index, IKES; Isometric knee extension strength, TUG; Timed up and go

**p<0.01

The following factors were associated with knee OA (Table 3): BMI (odds ratio [OR]: 1.54, 95% confidence interval [95%CI]: 1.31–1.81), IKES (OR: 0.70, 95% CI: 0.62–0.78), and TGS (OR: 0.86, 95% CI: 0.76–0.98).

**Table 3:** The results of multiple logistic regression analysis.

	Odds ratio (95% confidence interval)
**Age (years)**	0.94 (0.87–1.01)
**BMI (kg/m**^**2**^**)**	1.54 (1.31–1.81)**
**TGS (kg)**	0.86 (0.76–0.98)*
**IKES (kg)**	0.70 (0.62–0.78)**

Hosmer-Lemeshow test, p = 0.51; rate of accurate discrimination, 88.2%; dependent variables: control group = 0, OA group = 1.

OA; osteoarthritis, BMI; body mass index, TGS; toe grip strength, IKES; isometric knee extension strength

*p<0.05

**p<0.01

## Discussion

We identified an association between TGS and knee OA among Japanese women. To our knowledge, this is the first study to have reported a relationship between TGS and knee OA.

Participants in the OA group had a higher BMI and lower IKES than participants in the control group. The reduction in activity associated with knee OA might not only result in an increase in BMI and a decrease in IKES, but also a decrease in TGS, though we did not investigate the physical activity level of participants. Because many patients in our OA group consisted of patients awaiting TKA with a severe KL grade, they might have a long history of OA, which may have resulted in marked weight gain and a decrease in muscle strength; with BMI and IKES having a stronger effect on their current status than TGS. Meanwhile, a sub-analysis with a comparison between OA participants with KL2, KL3, and KL 4 and the control group revealed the same results (Table 1). Multiple logistic regression analysis did confirm TGS is a factor associated to knee OA, independent of age, BMI, and IKES (Table 3).

We previously identified a decrease in TGS with aging [24, 25, 29] as well as a significant positive correlation between TGS and IKES in healthy women [29]. These results are confirmed in our current study, both in our healthy control group and our knee OA group. Moreover, as in our previous study [29], TGS was significantly correlated to the TUG in our control group, but not in our OA group. Previous studies have reported a significant association between TGS and fast-paced walking [30, 31]. The absence of an association between TGS and the TUG in our OA group might reflect the slower gait speed that is typical in individuals with knee OA [32, 33]. The TUG in our OA group was significantly slower than for our control group and was, in fact, close to the suggested TUG cut-off value for identifying community-dwelling individuals at risk of falling [32, 34, 35]. In addition, correlations were greater for the control group than for the OA group, which may be associated with reduced physical activity resulting from knee OA as stated previously. Although we also considered the influence of knee pain or stiffness, TGS was not significantly correlated with pain and stiffness assessed by the WOMAC scale (data not shown).

Saito et al. [33] reported that partial foot pressure under the hallux was significantly lower in patients with knee OA than in healthy young and older adults during walking, with a shorter overall path of the center of pressure in the OA group than the healthy control group. Therefore, patients with knee OA might not bear sufficient weight on the toe region of the foot during walking [33]. Arnold et al. [36] further reported a decreased range in sagittal plane motion at the hallux in patients with knee OA compared to a healthy control group. Based on these reports, an association is inferred between toe function and knee OA. In addition, Hessert et al. [37] also identified decreased pressure under the hallux region and decreased sagittal plane range of motion at preferred walking speed as specific features of aging on the kinematics of the foot [36]. Therefore, although we could not evaluate a causal relationship between TGS and the progression of knee OA in our study design, we speculate there may be an association between decreasing TGS and the incidence of knee OA with aging.

We propose that age-related impairment in TGS and changes in the kinematics of the toes during gait might induce detrimental mechanical stress at the knee joint, leading to degenerative change in the articular cartilage of the knee. Our previous research demonstrated that decreased pressure under the toes during gait, described as “gait with a floating toe”, generates larger vertical acceleration of the knee compared to gait with normal foot kinematics in an experimental setting [38]. In the current study, measured TGS was strongly associated with the strength of the hallux, based on the testing methods used [24–26], which likely explains the identification of TGS as an independent factor associated with knee OA revealed in our multiple regression analysis. The association between impairments in the function of the hallux and knee OA is also supported by Dananberg [20, 21] who demonstrated that decreased extension of the hallux during gait produced a compensatory flexion of the knee. As knee flexion decreases the joint contact area [39], prolonged knee flexion, even at small angles, would produce potentially detrimental mechanical stress on the articular cartilage of the knee, with cumulative mechanical stress on the tibiofemoral joint resulting in the development of OA. In contrast, the kinematics of the foot, including the pressure distribution under the foot, is dependent on the speed of walking [36]. A reduced walking speed, which is commonly observed in patients with knee OA [33], decreases the motion of the hallux [40] and the partial pressure developed under it during terminal stance [33], with an overall shorter path of the center of pressure under the foot [33]. These changes might lead to a decrease in TGS or, conversely, a decrease in TGS could lead to these changes and the development or progression of knee OA. Regardless of the causal path, there is an interdependence between TGS and knee OA that can result in negative changes in both factors.

The limitations of our study need to be acknowledged. Foremost, the majority of patients in our OA group [40] had severe OA (53.3% with a KL grade of 4 and 10% with a grade of 2), with 104 of the 120 patients in the OA group being hospitalized as they awaited TKA. Thus, patients in our OA group might have a long history of OA, which may have resulted in marked weight gain and decrease in muscle strength, with BMI and IKES having a stronger effect on their current status than TGS. Future studies will need to include more patients with mild knee OA. Second, individuals forming our control group were independent in the community and participated in community-based sports event. Therefore, these individuals were likely to be health conscious and, as such, their physical fitness and muscle strength were likely better than in average community dwelling older adults. Meanwhile, we could not consider the influence of physical activity levels on the results of this study because physical activity levels of participants were not evaluated. As well, we only included women in our study to control for sex-specific differences in the prevalence of knee OA and gait kinematics. Therefore, the relationship between TGS and knee OA in men remains to be evaluated. Lastly, we conducted a cross-sectional observational study and, therefore, longitudinal and interventional studies are needed to clarify the causal relationship between foot function and knee OA.

## Conclusions

We describe a probable relationship between TGS and knee OA in older Japanese women. Based on our results, we propose that physiotherapy intervention to strengthen TGS might be a practical method to have some influence on the occurrence or progression of knee OA pathology.

## Supporting information

S1 Dataset S1_Dataset. (XLSX)
